# Protein-observed 19F NMR of LecA from *Pseudomonas aeruginosa*

**DOI:** 10.1093/glycob/cwaa057

**Published:** 2020-07-01

**Authors:** Elena Shanina, Eike Siebs, Hengxi Zhang, Daniel Varón Silva, Ines Joachim, Alexander Titz, Christoph Rademacher

**Affiliations:** 1 Max Planck Institute of Colloids and Interfaces, Department of Biomolecular Systems, Am Mühlenberg, 14424 Potsdam, Germany; 2 Free University of Berlin, Department of Biochemistry and Chemistry, 14195 Berlin, Germany; 3 Chemical Biology of Carbohydrates, Helmholtz Institute for Pharmaceutical Research Saarland, Helmholtz Centre for Infection Research, 66123 Saarbrücken, Germany; 4 Saarland University, Department of Pharmacy, 66123 Saarbrücken, Germany; 5 German Center for Infection Research, Hannover-Braunschweig, Germany

**Keywords:** drug discovery, LecA, lectin, NMR

## Abstract

The carbohydrate-binding protein LecA (PA-IL) from *Pseudomonas aeruginosa* plays an important role in the formation of biofilms in chronic infections. Development of inhibitors to disrupt LecA-mediated biofilms is desired but it is limited to carbohydrate-based ligands. Moreover, discovery of drug-like ligands for LecA is challenging because of its weak affinities. Therefore, we established a protein-observed 19F (PrOF) nuclear magnetic resonance (NMR) to probe ligand binding to LecA. LecA was labeled with 5-fluoroindole to incorporate 5-fluorotryptophanes and the resonances were assigned by site-directed mutagenesis. This incorporation did not disrupt LecA preference for natural ligands, Ca^2+^ and d-galactose. Following NMR perturbation of W42, which is located in the carbohydrate-binding region of LecA, allowed to monitor binding of low-affinity ligands such as *N*-acetyl d-galactosamine (d-GalNAc, *K_d_* = 780 ± 97 μM). Moreover, PrOF NMR titration with glycomimetic of LecA *p*-nitrophenyl β-d-galactoside (pNPGal, *K_d_* = 54 ± 6 μM) demonstrated a 6-fold improved binding of d-Gal proving this approach to be valuable for ligand design in future drug discovery campaigns that aim to generate inhibitors of LecA.

## Introduction

Many opportunistic pathogens, such as the Gram-negative bacterium *Pseudomonas aeruginosa*, use glycan-binding proteins (lectins) to infect the host and to establish a protective antibiotic-resistant biofilm environment in the lungs of immunocompromised patients (Singh et al. 2000). LecA plays a key role in this process and has become a promising target to prevent biofilm formation and consequently disease progression (Diggle et al. 2006; Wagner et al. 2016).

**Fig. 1:**
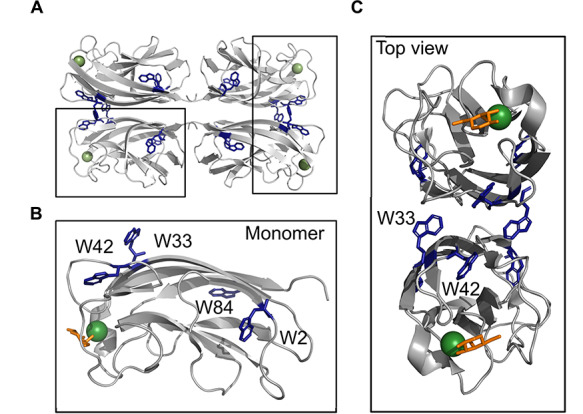
Structure of LecA. Cartoon representation of tetramer LecA (PDB: 4CP9). Shown is an expansion of a LecA monomer with D-Gal (shown as sticks), Ca^2+^ ion (shown as sphere) and positions of four tryptophanes (W2, W33, W42, W84). W2 and W84 are in the protein core. Top view shows W42 and W33 being located near the carbohydrate-binding site of LecA.

LecA forms a protein homotetramer (Fig. 1A) and requires a calcium (II) ion (Ca^2+^) to coordinate binding to its natural monosaccharide ligand, d-galactose (d-Gal) (Cioci et al. 2003). Notably, each monomer has four tryptophan residues (Fig. 1B), where W42 and W33 reside in close proximity to the d-Gal-binding region and near the hinge connecting the two domains, respectively (Fig. 1C). In the physiological context of a biofilm, the low micromolar affinity of LecA for d-Gal (*K*_d_ = 88 μM, (Kadam et al. 2011)) is compensated with high avidity of ligands that can crosslink the neighboring carbohydrate-binding sites, such as GalAxG3 (*K*_d_ = 2.5 nM, (Bergmann et al. 2016; Cecioni et al. 2015)); however, drug-like molecules do not benefit from such multivalency. Successful approaches to find low-molecular-weight inhibitors have capitalized on the β-linked galactosides with aromatic aglycon (Garber et al. 1992), such as *p*-nitrophenyl *β-*d-galactoside (pNPGal) or GalAG0 with *K_d_* = 14.1 μM and *K_d_* = 4.2 μM, respectively (Kadam et al. 2011; Kadam et al. 2013; Rodrigue et al. 2013). This improvement in binding affinity is due to the additional CH–π interaction between the aromatic ring of the binding ligand and H50 at the binding pocket. Cumulatively, development of inhibitors for LecA consists of β-linked carbohydrate-based ligands (glycomimetics, (Wagner et al. 2017)) and, therefore, having drug-like ligands for LecA is crucial in future research, but discovery of such weak binders is challenging. Therefore, new methods to detect binding of weak ligands to LecA are required.

Biophysical methods are suitable to identify low-molecular-weight and low-affinity ligands (Renaud et al. 2016). 19F nuclear magnetic resonance (NMR) has proven to be valuable in the study of protein–ligand interactions for several reasons. The isotope 19F 1) has the spin 1/2 nucleus and a natural abundance of 100%, 2) is very stable and 3) nearly absent in biological systems delivering a background-free NMR spectrum (Luck and Falke 1991). In the case of 19F-labeled proteins used in protein-observed 19F (PrOF) NMR, the size of protein is not a limitation and the protein side chains are detected as broad resonances at low (25 μM) to mid-micromolar (200 μM) protein concentrations (Kitevski-LeBlanc and Prosser 2012; Liu et al. 2012). This method has benefited from the commercial availability of many fluorinated aromatic amino acids, such as 5-fluorotryptophan (5FW), 3-fluorotyrosine and 4-fluorophenylalanine. Unfortunately, these fluorine-labeled amino acids are expensive. In contrast, fluorine-labeled precursors of amino acids, such as 5-fluoroindole (5FI), can be employed to incorporate fluorine-labeled amino acids in proteins, resulting in reduced costs (Gee et al. 2016). Moreover, incorporation of fluorinated amino acids does not lead to major structural and functional perturbations (Arntson and Pomerantz 2016; Kitevski-LeBlanc and Prosser 2012; Sharaf and Gronenborn 2015). In this context, 5FW has been shown to have only a minor impact on protein structure and dynamics in bacterial lectin from *Ralstonia solanacearum* lectin (RSL) (Tobola et al. 2018).

Here, we explored PrOF NMR using LecA labeled with 5FW (5FW LecA) to detect binding of ligands with moderate as well as low affinities. To assign 5FW resonances, we produced its wild-type (WT) and four tryptophan-to-phenylalanine mutants (W2F, W33F, W42F and W84F). In the binding studies, we determined the dissociation constants of 5FW LecA with its natural ligands Ca^2+^, d-Gal and d-GalNAc. We compared the affinity data of LecA and 5FW LecA with other orthogonal biophysical methods, such as isothermal titration calorimetry (ITC) or competitive binding by fluorescence-polarization (FP) detection. Finally, we verified the suitability of 5FW LecA PrOF NMR for a ligand design using glycomimetics pNPGal and phenyl-β-d-galactopyranoside (Ph-β-d-Gal, (Imberty et al. 2004)).

**Fig. 2:**
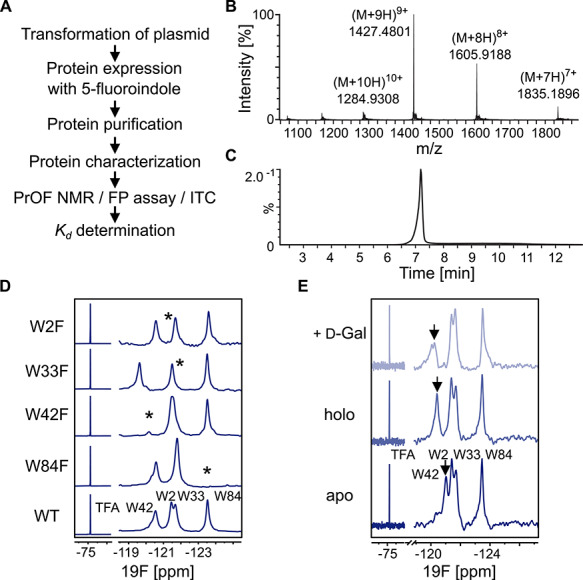
PrOF NMR of 5FW LecA. **(A)** General workflow for PrOF NMR with 5FW LecA. (**B)** Chromatogram of the LC–ESI–MS analysis of 5FW LecA. **(C)** ESI-MS^+^ spectrum of the main peak at 7.3 min [M + H]^+^Ca = 12826.23 Da [M + H]^+^found = 12831.34 Da corresponds to 5FW LecA. (**D)** PrOF NMR assignment of 5FW LecA WT and the mutants W84F, W42F, W33F and W2F. The tryptophanes being mutated are indicated with asterisk. All spectra were normalized and referenced to TFA. (**E)** PrOF NMR of 5FW LecA WT in Ca^2+^-free (apo, *bottom*) and -bound (holo, *central*) forms. The W42 resonance (*black arrow*) shifted in presence of Ca^2+^ and 0.5 mM d-Gal binding verifying that protein is active.

## Results and discussion

### Protein expression and characterization

For the stable incorporation of 5FW in LecA we followed the workflow shown in Fig. 2A. *Escherichia coli* BL21 (DE3) cells were grown in presence of 5FI and the protein was characterized for fluorine incorporation *via* mass spectrometry (Fig. 2B and C). In the mass spectrum 5FW LecA had a dominant mass of 12831.34 Da corresponding to full incorporation of four tryptophan residues being replaced with 5FW. Protein yields as high as 45–50 mg L^−1^ using non-auxotrophic *E. coli* BL21 (DE3) cells were achieved. This compares very well to protein expression yields under non-labeling conditions (30–35 mg L^−1^).

### Establishing 5FW LecA in PrOF NMR

To optimize PrOF NMR for 5FW LecA, we varied temperature (Supplementary Fig. S1), protein concentration (Supplementary Fig. S2) and buffer (Supplementary Fig. S3). Fluorine resonances of 5FW LecA resulted in a well-resolved PrOF NMR spectrum using 100–200 μM 5FW LecA at 310 K on a 700 MHz NMR machine equipped with a cryogenic probe (Supplementary Table SI).

Next, we assigned the fluorine resonances in PrOF NMR to four tryptophanes of LecA using site-directed mutagenesis (Fig. 2D). The disappearance of fluorine resonances in LecA mutants being replaced with phenylalanine gave indication for the assignment. As result, two peaks at −120.47 and −123.24 p.p.m. could be reliably identified as W42 and W84, respectively. Two resonances at −121.43 and −121.72 p.p.m. overlapped slightly indicating that both reside in a similar chemical environment in LecA, but could be assigned as W2 and W33, respectively.

### Incorporation of 5FW into LecA does not abrogate protein activity

Because in some cases the affinity of proteins to the carbohydrates might be changed due to 5FW incorporation, as it has been shown for a stronger affinity of 5FW to β-d-Gal (Hudson et al. 2015), we confirmed that incorporation of 5FW in LecA does not introduce structural or functional perturbations that could alter protein activity. For this, we measured 5FW LecA in PrOF NMR in its Ca^2+^-free (apo), -bound (holo) and d-Gal-bound forms (Fig. 2E). As the preference of LecA for both Me-α-d-Gal and Me-β-d-Gal has been reported (*K_d_* = 50 μM and 55.7 μM, respectively; Rodrigue et al. 2013), suggesting that the anomeric configuration of d-Gal is not critical, we used d-Gal containing both anomeric configurations to verify the protein activity. As a result, 19F resonance of W42 showed a chemical shift perturbation (CSP) upon binding to both Ca^2+^ and d-Gal indicating that 5FW LecA remained in its active form.

**Fig. 3:**
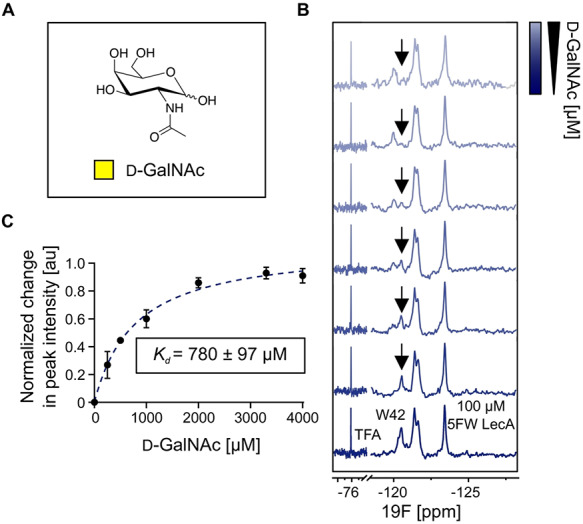
PrOF NMR to probe weak 5FW LecA–ligand interactions. (**A)** Structure of *N*-acetyl d-galactosamine (d-GalNAc). (**B)** The PrOF NMR spectra of holo 5FW LecA (*bottom*) and titration of d-GalNAc (*upper*). d-GalNAc binding affects W42 the strongest and thus, we followed the changes in peak intensity of free W42 to derive *K_d_* values for d-GalNAc binding. (**C)** Binding isotherm for d-GalNAc generated by plotting the normalized change in peak intensity of 5FW free W42 resonance as a function of ligand concentration. Data of three independent titrations were fitted to one-site-binding model to obtain *K_d_* of 780 ± 97 μM.

Next, we measured the affinities of 5FW LecA to its natural ligands. For this, we titrated Ca^2+^ (Supplementary Fig. S4) and d-Gal (Supplementary Fig. S5) to 5FW LecA, which resulted in binding affinities *K_d_* of 47$\pm$8 μM and 360$\pm$47 μM, respectively. Despite the difference to previously reported affinity for d-Gal (Kadam et al. 2011), the 2- or 3-fold deviation in binding affinities determined in PrOF NMR has been considered acceptable in PrOF NMR (Gee et al. 2016; Tobola et al. 2018). In our experience, we have considered a 4-fold change acceptable to continue with affinity assessment.

Next, we confirmed the affinities for Ca^2+^ and d-Gal with both LecA and 5FW LecA in ITC (Supplementary Fig. S6) and a competitive binding fluorescence polarization (FP) assay, respectively (Supplementary Fig. S7; Joachim et al. 2016). As a result, binding experiments of 5FW LecA with Ca^2+^ and d-Gal confirmed the affinities to be in similar range with LecA (Supplementary Table SIV), concluding that 5FW LecA preserved its activity and preference to its natural ligands similarly to LecA.

### PrOF NMR to probe weak LecA–ligand interactions

To establish a method for the discovery of drug-like molecules for LecA, our aim was to probe 5FW LecA in PrOF NMR for binding of a known weak ligand. For this, we chose d-GalNAc (Fig. 3A; Chemani et al. 2009). We observed that d-GalNAc perturbed W42 resonance located in the carbohydrate-binding site of 5FW LecA. The changes in W42 peak intensity (Fig. 3B) upon addition of d-GalNAc were followed to derive the *K_d_* value of 780$\pm$97 μM (Fig. 3C).

Similarly as before, we compared the affinities of 5FW LecA for d-GalNAc in a FP-based assay and the IC_50_ was 3-fold higher compared with the *K_d_* obtained from PrOF NMR confirming that d-GalNAc is much weaker ligand compared with Ca^2+^ or d-Gal. Moreover, our affinity data in the FP assay for ligands, in particular d-Gal, were in a close range 1230 ± 200 μM and 1991 μM for both unlabeled LecA and 5FW LecA, respectively (Supplementary Table SIV). Cumulatively, this result suggests that the affinities for d-GalNAc derived from the FP assay for LecA and 5FW LecA diverged from PrOF NMR because of higher sensitivity of 19F NMR to spot weak binders and thus, thereby shows the advantages of PrOF NMR in discovery of weak interactions.

### 5FW LecA PrOF NMR is sensitive to probe glycomimetics

**Fig. 4:**
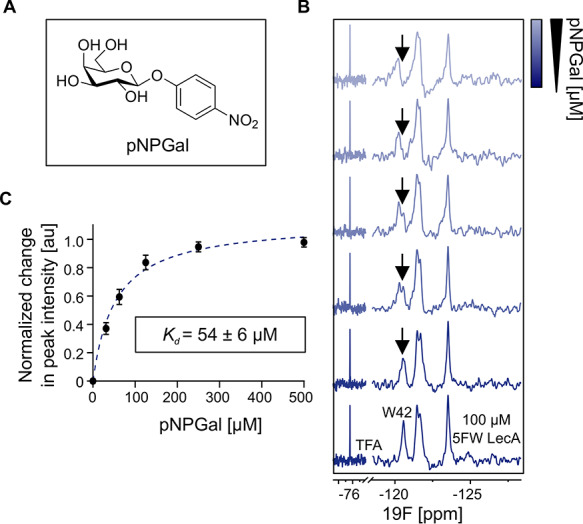
PrOF NMR titration of a carbohydrate-based glycomimetic pNPGal to holo 5FW LecA. **(A)** The structure of pNPGal. **(B)** The PrOF NMR spectra of holo 5FW LecA (*bottom*) and titration of pNPGal (*upper*). The peak intensity of W42 resonance (*arrow*) decreased upon pNPGal addition. The change in signal intensity of free W42 peak can be followed to determine *K_d_*. (**C)** Binding isotherm for pNPGal generated by plotting the normalized change in peak intensity of 5FW free W42 resonance as a function of ligand concentration. Data of three independent titrations were fitted to one-site-binding model to obtain *K_d_* of 54 ± 6 μM.

PrOF NMR with 5FW can be useful for discovery and design of ligands for LecA. For this, we performed PrOF NMR titrations of two glycomimetics: phenyl-Ph-β-d-Gal (Supplementary Fig S6) and pNPGal (Fig. 4) to 5FW LecA resulting in *K_d_* of 166 ± 42 μM and 54 ± 6 μM, respectively. Moreover, p-nitrophenyl group improved binding affinity of d-Gal 6-fold, which is in agreement with previous reports (Rodrigue et al. 2013). This shows that 5FW in LecA can serve as sensitive probes to follow the affinity gain to design glycomimetics using structure-activity relationship approach (Divakaran et al. 2019).

## Conclusions

We have shown that 5-fluoroindole can be used as a precursor of 5FW to label LecA for PrOF NMR studies. In our binding studies with Ca^2+^, d-Gal and d-GalNAc, PrOF NMR has proven to detect and determine the affinity of moderate as well as weak ligands. In contrast to ligand-observed NMR techniques (e.g. STD NMR; Mayer and Meyer 2001) providing information on the epitope of ligand binding, PrOF NMR provides information on the ligand-binding site in the protein.

Further studies using ITC and FP assays have demonstrated that 5FW LecA preserved its activity and the ligand preference similarly to LecA. Notably, PrOF NMR has proven more sensitive for identification of weak ligands like d-GalNAc due to chance to observe the formation of a protein–ligand complex in NMR at earlier time point compared with the FP assay. Accordingly, these results represent the first studies demonstrating the potential of 5FW LecA PrOF NMR to assess binding of weak ligands. As tryptophan is by far the most frequently found amino acid in carbohydrate binding sites of various lectins (Taroni et al. 2000), this method could prove to be a valuable tool to assess binding of fragment- and drug-like molecules targeting the carbohydrate binding site of various lectins. Together, this approach will support the future drug-discovery campaigns that aim to develop drug-like inhibitors for lectins such as LecA.

## Materials and methods

### Fluorinated protein expression and purification

Recombinant 5FW LecA (WT and mutants) was expressed and purified as follows: *E. coli* BL21 (DE3) cells were transformed with pET25pa1l plasmid and grown in LB medium (100 μg mL^−1^ ampicillin) at 37°C with agitation (120 rpm) until OD_600_ reached 0.6. 1 L of culture was harvested by centrifugation at 2500 × g, 10 min and resuspended in modified minimal M9 medium (Supplementary Table SIII). It was shaken at 37°C for 60 min as a recovery time for bacteria followed by addition of 250 μL of 5-fluoroindole (Santa Cruz, USA; 240 mg/mL in dimethyl sulfoxide [DMSO]). Protein production was induced with 250 μM IPTG at 30°C and harvested in 4 h. Cell pellets were resuspended in buffer A (20 mM Tris-HCl pH 7.4, 137 mM NaCl, 2.6 mM KCl, 25 mM CaCl_2_) supplemented with 1 mM PMSF and DNaseI (Applichem, Darmstadt, Germany). The cells were lysed by cell disruption (Branson Digital Sonifier) at 50% power 10 s on and 40 s off pulses following removal of cell debris by centrifugation (10,000 × g, 30 min, 4°C). The supernatant was loaded onto a 2 mL Pierce™ d-Gal agarose column (Thermo Fisher Scientific) that was equilibrated with 3-fold column volume of buffer A. Bound LecA was eluted with buffer B (20 mM Tris-HCl pH 7.4, 137 mM NaCl, 2.6 mM KCl, 25 mM CaCl_2_, 100 mM d-Gal). Protein was dialyzed in MilliQ water and TBS buffer (20 mM Tris-HCl pH 7.8, 100 mM NaCl) or MES buffer (25 mM MES pH 6, 40 mM NaCl) three times for 4 h and once overnight at 4°C, respectively. The protein solution was flash frozen and stored at −80°C.

### Protein-observed fluorine (PrOF) NMR of 5FW LecA

All experiments were conducted on Bruker Ascend™700 (AvanceIII HD) spectrometer equipped with a 5 mm TCI700 CryoProbe™ in 3 mm tubes (Norell S-3-800-7) with following parameters: time domain of 1972, relaxation delay 1 s, acquisition time of 0.15 s, spectral width of 10 p.p.m. and 1024 scans resulting in measurement time of 20 minutes.

For optimization, PrOF NMR with 50, 100 or 200 μM holo 5FW LecA was recorded in TBS pH 7.8 or MES pH 6 with 10% D_2_O, 2 mM CaCl_2_ and 100 μM TFA at 285, 298 or 310 K. We considered only changes in CSP of peaks being 2-fold greater than standard deviation of fluorine resonance upon addition of 10 mM CaCl_2_ or 1 mM d-Gal. All data analysis, plotting and curve fitting were performed with MestReNova 11.0.0 (Mestrelab Research SL, Santiago de Compostela, Spain). All spectra were referenced and normalized to trifluoroacetic acid (TFA) as internal reference at −75.6 p.p.m. after applying the Exponential function (30 Hz) and baseline correction.

The PrOF NMR titrations of d-Gal, d-GalNAc, Ph–β-d-Gal and pNPGal were performed with 100 μM 5FW LecA in TBS pH 7.8 at 310 K. For Ca^2+^ titration, 5FW LecA was dialyzed against Chelex®-100 in MES pH 6 buffer at 4°C overnight.

The decreasing intensity of the unbound W42 in 5FW LecA was followed to determine *K_d_* values of ligands. Here, we used these values to normalize the changes in W42 peak intensities (*I*_normalized_) following the equation (1) resulting in values plotted on Y-axis.

(1) \begin{equation*} {I}_{\mathrm{normalized}}=\frac{I_0-{I}_{\mathrm{measured}}}{I_0}, \end{equation*}

where *I_0_* was unbound W42 in the reference spectrum of protein only, *I*_measured_ was unbound W42 peak of protein with a ligand. The *K_d_* values were calculated according to the one-site-binding model in GraphPad Prism 8 (GraphPad Software Inc., San Diego, CA) from three independent titrations.

### Isothermal titration calorimetry

ITC was performed on a Microcal ITC200 (General Electric) at 25°C. Calcium ions were removed by extensive dialysis against 1 mM EDTA pH 7.4 (×4) followed by 150 mM NaCl (×4) and distilled water. The protein solution was lyophilized and the solid protein stored at −20°C. A solution of calcium chloride in TBS (20 mM Tris, 137 mM NaCl, 2.6 mM KCl at pH 7.4) was titrated into a calcium-free LecA solution in the same buffer. The data were analyzed according to the one-site-binding model using Microcal Origin software. Four independent titrations were performed using CaCl_2_ and LecA between 2 and 3 mM and 170 and 200 μM, respectively.

### Competitive binding fluorescence polarization assay

The competitive binding assay was performed as reported previously (Joachim et al. 2016). In total, 10 μL of LecA (40 μM) and *meta*-linked fluorescein-conjugate of phenyl-galactopyranoside (20 nM) in TBS pH 7.4 supplemented with 1 mM CaCl_2_ (TBS/Ca^2+^) and 10 μL of compound-dilution series (8 mM to 62 μM, 8% DMSO) in TBS/Ca^2+^ buffer were mixed in a 384-well plate (Greiner Bio-One, Germany) in three technical replicates. The sealed plate was centrifuged at 300 g for 1 min and incubated at room temperature with shaking. Fluorescence intensity was measured on a PheraStar FS microplate reader (BMG Labtech GmbH, Germany; ex. 485, em. 535 nm) after 1 and 16 h. Polarization was calculated and the data were analyzed according to the four-parameter variable slope model (MARS Data Analysis Software, BMG Labtech GmbH, Germany), the top and bottom plateaus were determined from the control Me-α-d-Gal and data were reanalyzed with these values fixed.

## Supplementary Material

Supplementary_information_revised_cwaa057

GLYCO-2020-00049_Revision_cwaa057
