# Supplementary material for: Protein-observed 19F NMR of LecA from Pseudomonas aeruginosa
Source: Glycobiology. 2020 Jul 1;31(2):159–65. doi: 10.1093/glycob/cwaa057 (PMC7874386; doi:10.1093/glycob/cwaa057)
Supplement: Supplementary_information_revised_cwaa057 [file supplementary_information_revised_cwaa057.pdf]

Supplementary data

Protein-observed 19F NMR of LecA from *Pseudomonas aeruginosa*

Elena V. Shanina[1,2], Eike Siebs[3,4,5], Hengxi Zhang[1,2], Daniel Varón Silva[1,2], Ines Joachim[3,4,5], Alexander Titz[3,4,5] & Christoph Rademacher[1,2,*]

[1] Max Planck Institute of Colloids and Interfaces, Department of Biomolecular Systems, Am Mühlenberg 14424, Potsdam, Germany;

[2] Free University of Berlin, Department of Biochemistry and Chemistry, 14195 Berlin, Germany;

[3] Chemical Biology of Carbohydrates (CBCH), Helmholtz Institute for Pharmaceutical Research Saarland (HIPS), Helmholtz Centre for Infection Research, 66123 Saarbrücken, Germany;

[4] Saarland University, Department of Pharmacy, 66123 Saarbrücken, Germany;

[5] German Center for Infection Research (DZIF), Hannover-Braunschweig, Germany.

Table of contents

Supplementary materials and methods

Site−directed mutagenesis

Liquid chromatography−mass spectrometry (LC−MS)

Materials

Supplementary tables

Table SI. Line width values of four tryptophan residues measured in PrOF NMR upon variation of buffer, temperature or protein concentration.

Table SII. Primer sequences used for single−point mutagenesis.

Table SIII. List of components used to prepare minimal medium (M9) supplemented with an amino acid cocktail and 5−fluoroindole (5FI).

Table SIV. *PrOF NMR and FP assay affinity values*

Supplementary figures

Fig. S1. Temperature optimization for 5FW LecA PrOF NMR**.**

Fig. S2. Optimization of 5FW LecA concentration for PrOF NMR**.**

Fig. S3. Buffer optimization of 5FW LecA for PrOF NMR**.**

Fig. S4. PrOF NMR titration of Ca^{2+} to apo 5FW LecA.

Fig. S5. PrOF NMR titration of D-Gal to holo 5FW LecA.

Fig. S6. ITC titration of Ca^{2+} to apo LecA.

Fig. S7. Competitive binding assay based on fluorescence polarization with LecA and 5FW LecA.

Fig. S8. PrOF NMR titration of Ph−β−D−Gal to holo 5FW LecA.

Supplementary materials and methods

Materials

All chemicals and buffers used within this work were purchased from Sigma Aldrich (St. Louis, MO, USA) or Carl Roth (Karlsruhe, Germany) unless otherwise indicated.

The LecA−encoding gene from *P. aeruginosa* PAO1 cloned into pET25(b+) vector resulted in pET25pa1l plasmid and LecA was produced recombinantly as reported previously (Blanchard, et al. 2008).

Site−directed mutagenesis

To distinguish fluorine resonances, we mutated tryptophan (W) against phenylalanine (F) using Quick−Change Lightning Mutagenesis Kit (Agilent, Santa Clara, USA). We used primers shown in (**Table SII**) following the manufacturer's protocol to generate mutants W33F, W42F and W84F. The plasmids were sequenced to confirm the mutagenesis using primers for T7 promoter. The plasmid for W2F mutant was obtained from GenScript Biotech (USA).

Liquid chromatography−mass spectrometry (LC−MS)

LC−MS analysis of LecA was performed using an ESI Waters Xevo G2−XS mass spectrometer coupled to an Acquity H−class UPLC system. LC separation was done on an Acquity UPLC Protein BEH C4 column (21 x 50 mm, 1.7 µm) using a gradient from 10 to 70% of acetonitrile in water with 0.5% formic acid in 12 min. The MS analysis was performed in positive mode using a 1.5 kV capillary voltage. The data were analyzed using MassLynx 4.2 and Biopharmalynx 1.3 software.

Supplementary tables

Table SI. Line width values of four tryptophan residues measured in PrOF NMR upon variation of buffer, temperature and protein concentration (*ND* = line width could not be determined due to low signal-to-noise quality of spectra).

Peak	Line width [Hz]							
	Buffer		Temperature			Protein concentration		
	TBS pH 7.8	MES pH 6.0	285 K	298 K	310 K	50 µM	100 µM	200 µM
W42	180	148	*ND*	*ND*	153	*ND*	170	178
W2	172	172	*ND*	161	125	*ND*	152	159
W33	161	144	*ND*	150	145	*ND*	167	165
W85	115	113	*ND*	127	116	*ND*	109	109

Table SII. Primer sequences used for single-point mutagenesis (F = forward, R = reverse).

Primer	Sequence
LecA W33F F	CGTAACTGGCGAAACCGGCGGCGACGA
LecA W33F R	TCGTCGCCGCCGGTTTCGCCAGTTACGGA
LecA W42F F	CCTGCGGCCCGAATTTCTGGGTAGGTCCGTA
LecA W42F R	TACGGACCTACCCAGAAATTCGGGCCGCAGG
LecA W84F F	ATTGGGTGCAACGAAACGGAACAACC
LecA W84F R	GGTTGTTCCGTTTCGTTGCACCCAAT
T7 PROMOTOR	TAATACGACTCACTATATAGG
T7 TERMINATOR	GCTAGTTATTGCTCAGCGG

Table SIII. List of components used to prepare minimal medium (M9) supplemented with an amino acid cocktail and 5−fluoroindole (5FI).

**Component**	**Amount per 1 L expression**
Autoclaved MiliQ water	500 mL
5x Amino acid cocktail	250 mL
10x M9 salts	100 mL
20% D-glucose	20 mL
100x Trace elements	10 mL
100 mg mL^{-1} Ampicillin	1 mL
1 mg mL^{-1} Vitamins	1 mL
1 M $CaCl_2$	300 µL
1 M $MgSO_4$	4 mL
Prepare 5−fluoroindole (60mg) in 250 µL DMSO	
**Amino acids cocktail (5x)**	
* For 1 L of stock solution, dissolve one by one in 50 mL of distilled MiliQ water and combine in following order:	
**Component**	
Alanine	500 mg
Arginine	400 mg
Asparagine Monohydrate	400 mg
Aspartic acid	400 mg
Cysteine-HCl-H_2O	50 mg
Glutamine	400 mg
Glutamic acid	650 mg
Glycine	550 mg
Histidine	100 mg
Isoleucine	230 mg
Leucine	230 mg
Lysine-HCl	420 mg
Methionine	250 mg
Phenylalanine	130 mg
Proline	100 mg
Serine	2.1 g
Threonine	230 mg
Tyrosine	170 mg
Valine	230 mg
Sodium acetate	1.5 g
Succinic acid	1.5 g
Potassium phosphate (dibasic)	10.5 g
Adenine	500 mg
Guanosine	650 mg
Thymine	200 mg
Uracil	500 mg
Cytosine	200 mg

**M9 salt solution (10x)**	
* For 1 L stock solution, dissolve in 800 mL, adjust the pH to 7.2 with NaOH, autoclave for 15 min at 121°C. Use non-^{15}N labeled NH_4Cl.	
Na_2HPO_4-$2H_2O$	75.2 g
KH_2PO_4	30 g
NaCl	5 g
NH_4Cl	5 g
**Trace elements (10x)**	
* For 1 L stock solution, sterilize the solution over a 0.22 µm filter. Store in dark.	
EDTA	5 g
In 800 mL MQ, adjust pH to 7.5 with NaOH	
$FeCl_3$ (anhydrous)	498 mg
$ZnCl_2$	84 mg
0.1 M $CuCl_2$-$2H_2O$	765 µL
0.2 M $CoCl_2$-$2H_2O$	210 µL
0.1 M H_3BO_3	1.6 mL
1 M $MnCl_2$-$4H_2O$	8.1 µL
Vitamin stock (1000x)	
* For 50 mL stock solution, sterilize the solution over a 0.22 µm filter. Prepare 1 mL aliquots and store at -20°C.	
Biotin	50 mg
Thiamin-HCl	50 mg

Table SIV. PrOF NMR and FP assay affinity values. Competitive binding assay of Me−α−D−Gal, D−GalNAc and D−Gal with LecA and 5FW LecA based on fluorescence polarization are shown. IC_{50} values are given as averages of three independent (LecA) or three technical replicates (5FW LecA) and is a relative affinity value depending on the concentration of ligand (8 mM) used in this assay, which was required to reduce binding of Me−α−D−Gal to half of the uninhibited value. K_d values for Me−α−D−Gal, D−GalNAc, D−Gal, Ph−β−D−Gal and pNPGal are defined as half of binding sites of 100 μM 5FW LecA being bound and are given as average of three independent replicates (*ND* = not determined).

**Competitive binding assay**			**PrOF NMR**
	LecA<br>IC_{50} [μM]	5FW LecA<br>IC_{50} [μM]	5FW LecA<br>K_d [μM]
Me−α−D−Gal	140 ± 30	195	*ND*
D−Gal	330 ± 40	317	360 ± 50
D−GalNAc	1230 ± 200	1991	780 ± 97
Ph−β−D−Gal	*ND*	*ND*	166 ± 42
pNPGal	*ND*	*ND*	54 ± 6

Supplementary figures

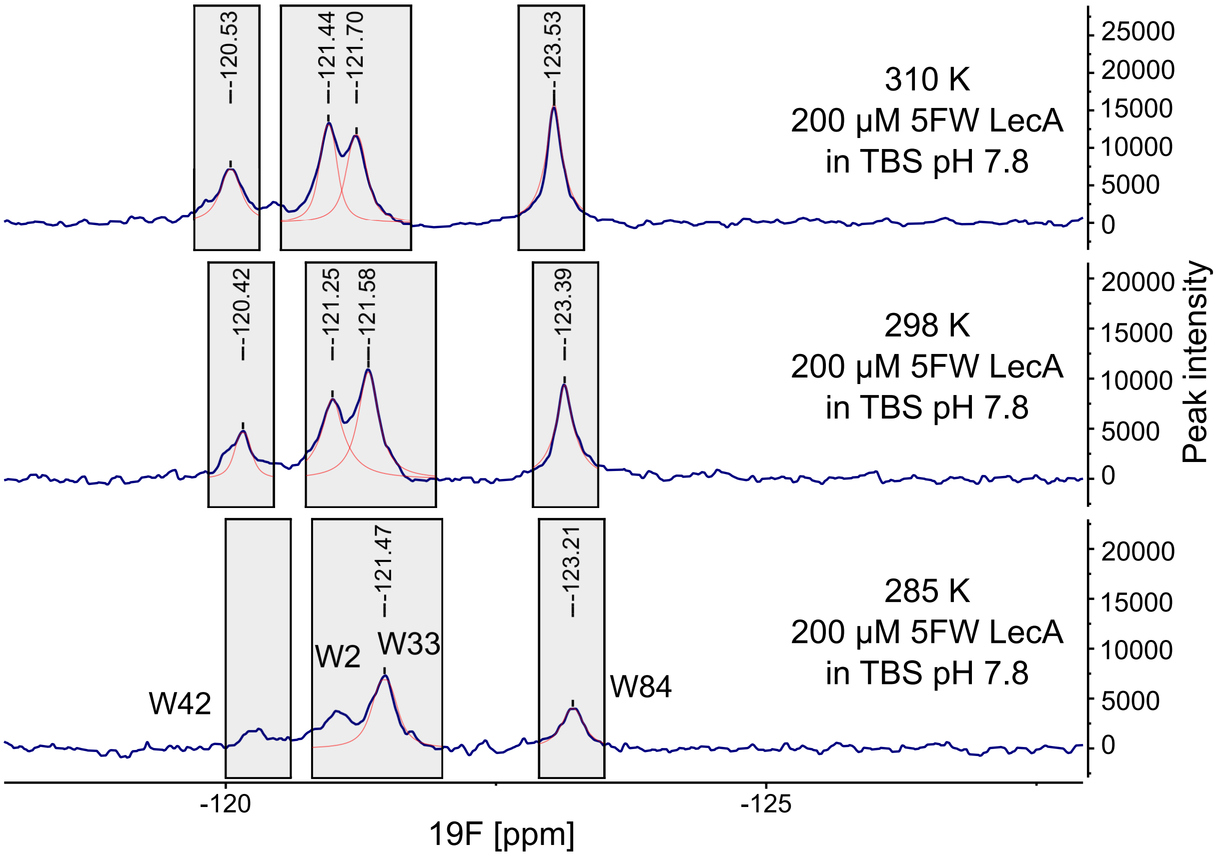

Fig. S1. Temperature optimization for 5FW LecA PrOF NMR**.** Shown is PrOF NMR spectrum of 200 μM holo 5FW LecA in TBS pH 7.8 at temperatures 285 K, 298 K and 310 K. Peak line widths of tryptophan resonances were measured in MestReNova using Line Fitting function for manual fitting of peaks (*red line*). The line width values for W42 and W84 at different temperatures are shown in **Table SI**, PrOF NMR spectrum of 5FW LecA at 298 K and 310 K resulted in similarly well-resolved spectra. The line width values of W2 and W33 deviated between measurements due to the overlap of both signals. As result, we used 310 K for PrOF NMR experiments with 5FW LecA**.**

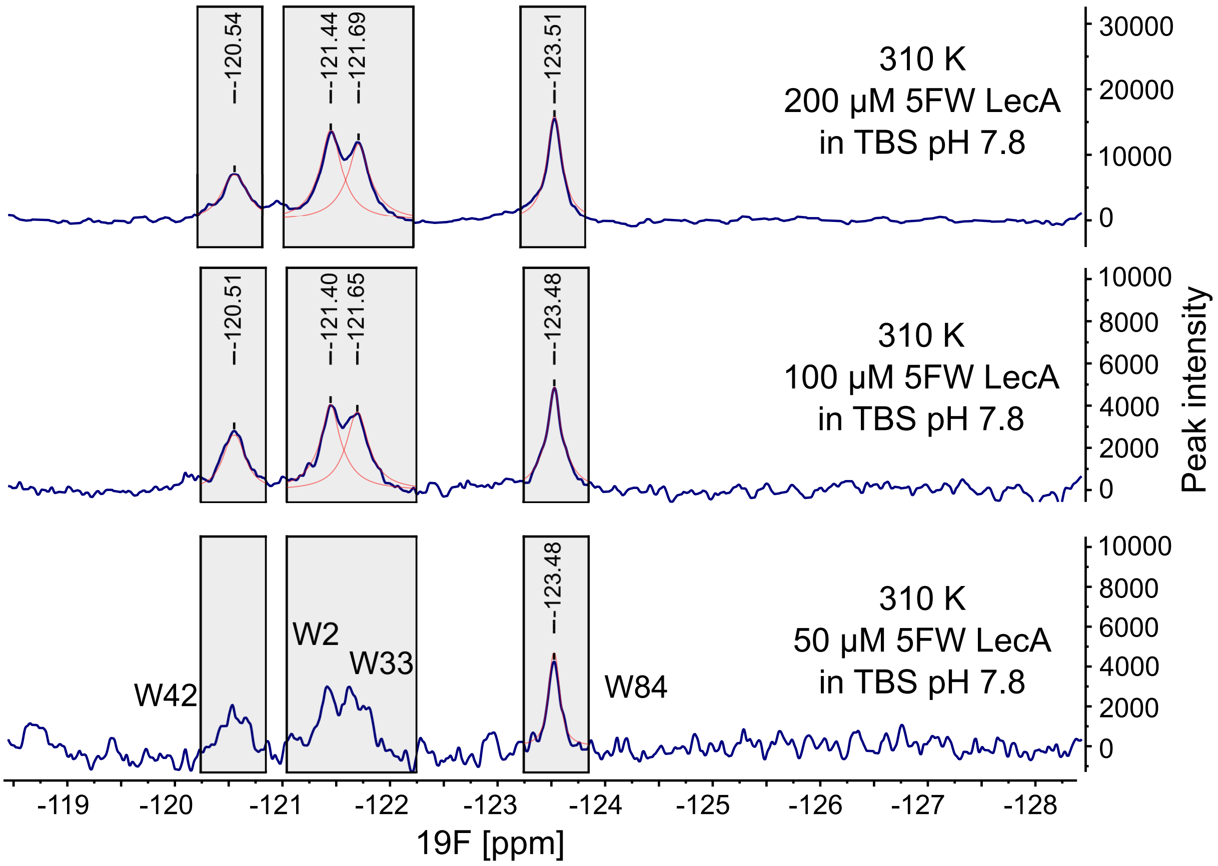

Fig. S2. Optimization of 5FW LecA concentration for PrOF NMR**.** Shown are PrOF NMR spectra of 50 µM (*bottom*), 100 µM (*middle*) and 200 µM (*top*) holo 5FW LecA in TBS pH 7.8 buffer at 310 K. Peak line widths of tryptophan resonances were measured in MestReNova x64 using Line Fitting function for manual fitting of peaks (*red line*). The line width values for W42 and W84 at concentrations 100 µM and 200 µM of 5FW LecA are shown in **Table SI**. Since PrOF NMR spectrum containing 50 µM 5FW LecA resulted in a low signal-to-noise spectrum, we did not measure line widths of tryptophan resonances. As result, 100 – 200 µM 5FW LecA concentrations can be used in future experiments.

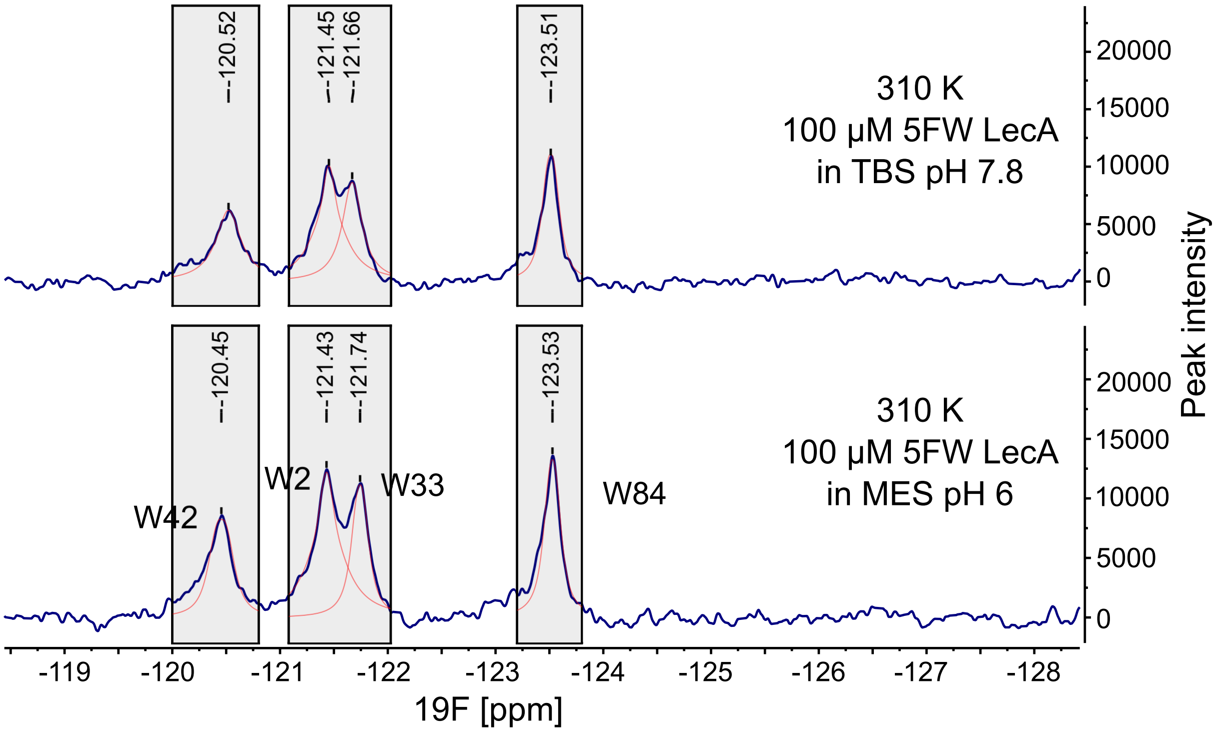

Fig. S3. Buffer optimization of 5FW LecA PrOF NMR**.** Shown is PrOF NMR spectrum of 100 μM holo 5FW LecA in a buffer with low (MES pH 6.0, *bottom*) *vs* medium salt (TBS pH 7.8, *top*) concentration. Both buffer systems show a comparable result on resolution of fluorine resonances**.** Peak line width values of tryptophan resonances were measured in MestReNova x64 using Line Fitting function for manual fitting of peaks (*red line*). The line width values for W42 and W84 are shown in **Table SI**, whereas line widths of W2 and W33 deviated between measurements due to the overlap of both signals.

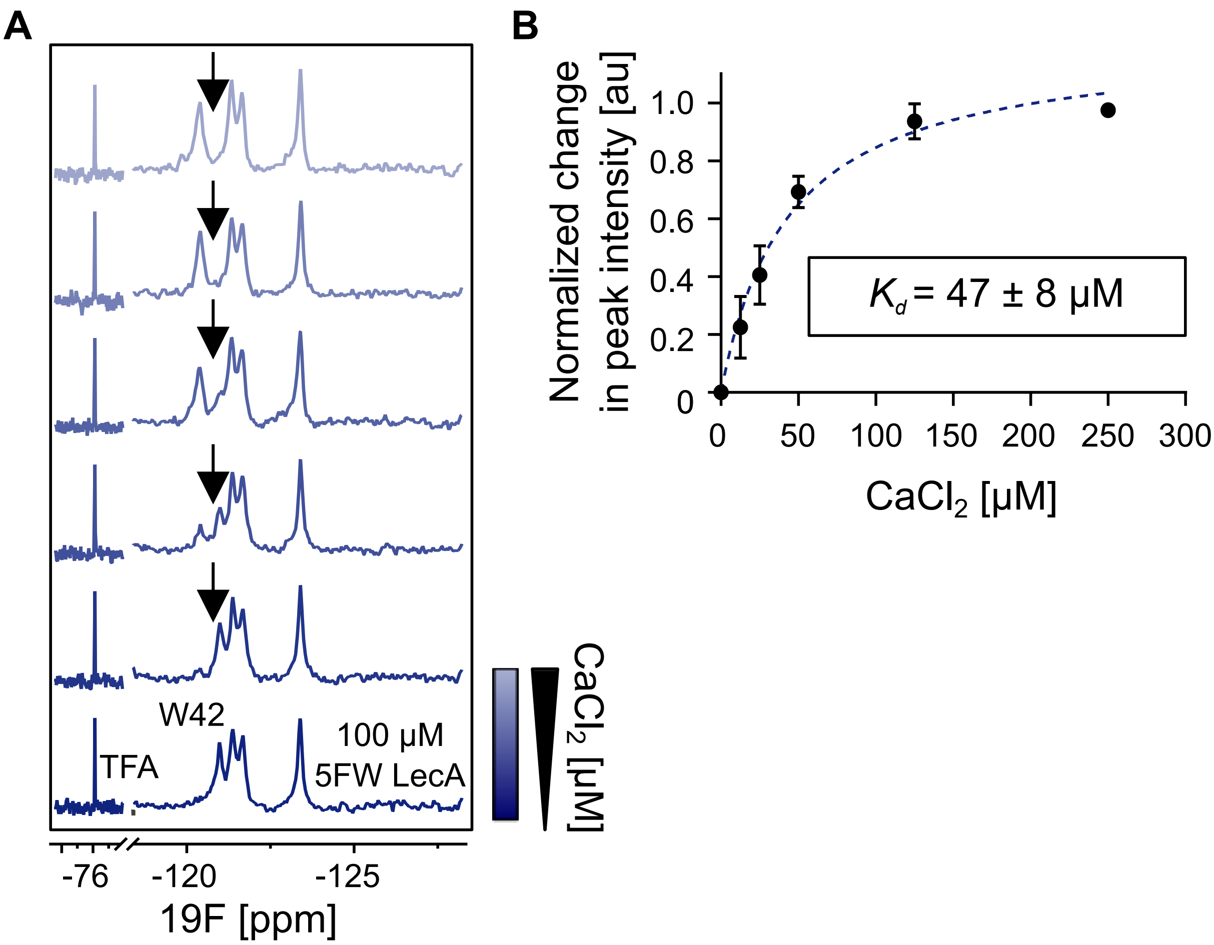

Fig. S4. PrOF NMR titration of Ca^{2+} to apo 5FW LecA. **A.** PrOF NMR titration of Ca^{2+} to Ca^{2+}−free (apo) 5FW LecA in MES pH 6 at 310 K. The W42 resonance undergoes a slow exchange on the chemical shift timescale and the signal intensity of Ca^{2+}−free W42 peak (*arrow*) can be followed to determine K_d. **B.** Shown is normalized change in unbound W42 peak intensity upon addition of Ca^{2+}. For this, the change in W42 peak intensity in presence of the Ca^{2+} compared to the reference was dived by W42 peak intensity of the reference spectrum delivering the normalized change in peak intensity values plotted on Y-axis. Titration data was fitted to one−site−binding model to obtain K_d value of 47±8 µM. The error bars represent the standard deviation of three independent titrations.

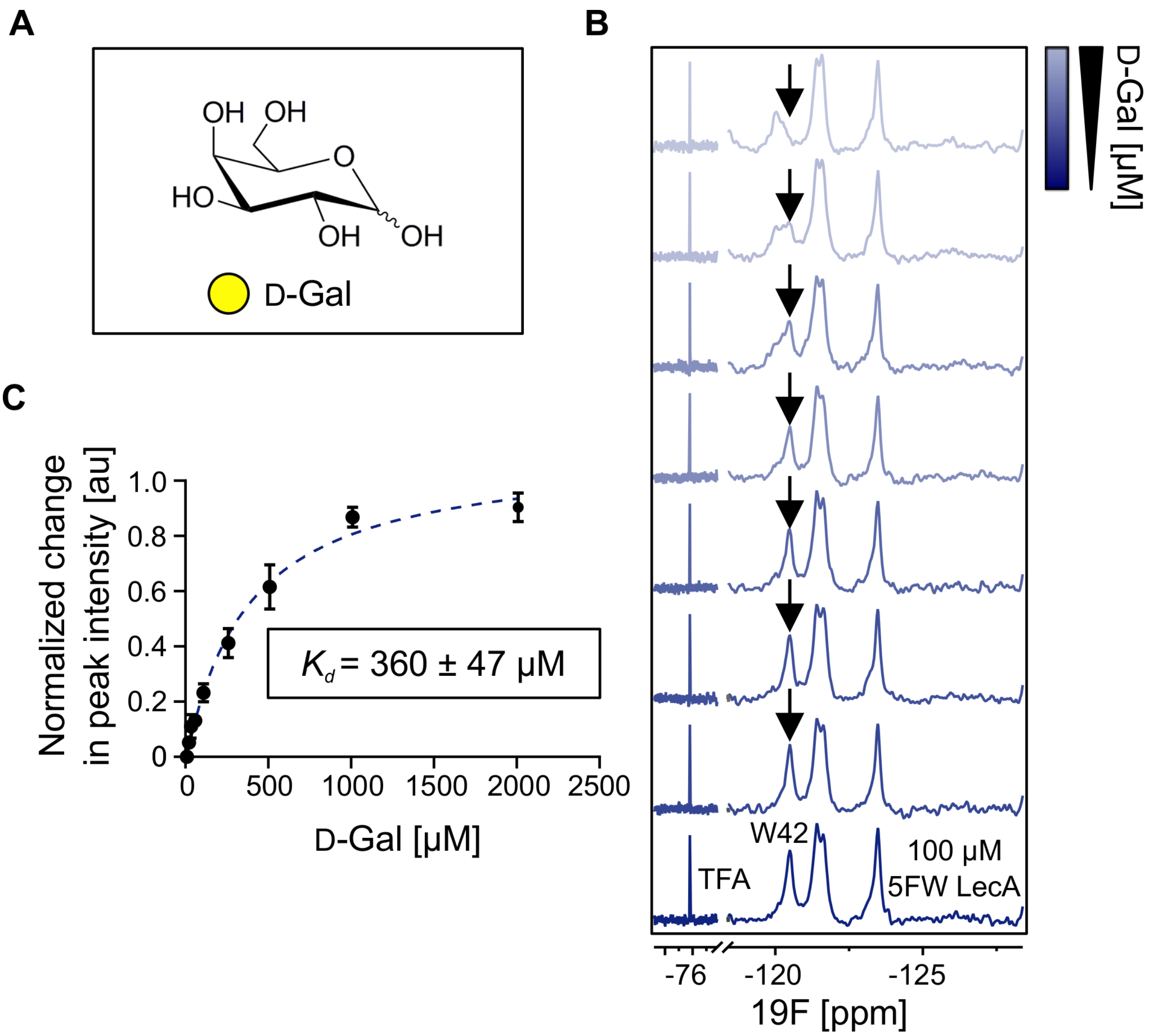

Fig. S5. PrOF NMR titration of D-Gal to holo 5FW LecA. **A.** The structure of D-Gal. **B.** PrOF NMR titration of D-Gal to holo 5FW LecA in TBS pH 7.8 at 310 K. The W42 resonance from free protein appeared with decreasing intensity (*arrow*) upon D-Gal addition and can be followed to determine K_d. **C.** Shown is normalized change in W42 peak intensity upon addition of D-Gal. For this, the change in W42 peak intensity in presence of the D-Gal was dived by W42 peak intensity of the reference spectrum delivering the normalized change in peak intensity values plotted on Y-axis. Titration data was fitted to one−site−binding model to obtain K_d value of 360 ± 47 µM. The error bars represent the standard deviation of three independent titrations.

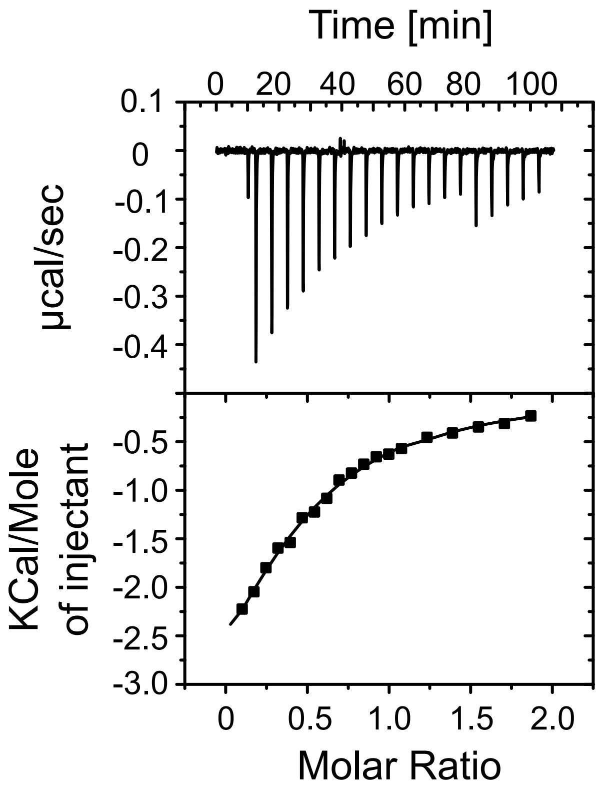

Fig. S6. ITC titration of Ca^{2+} to apo LecA. One representative graph of an isothermal titration microcalorimetry (ITC) of unlabeled LecA with Ca^{2+} is depicted. The K_d of 60±20 µM was determined from a minimum of three independent titrations.

A

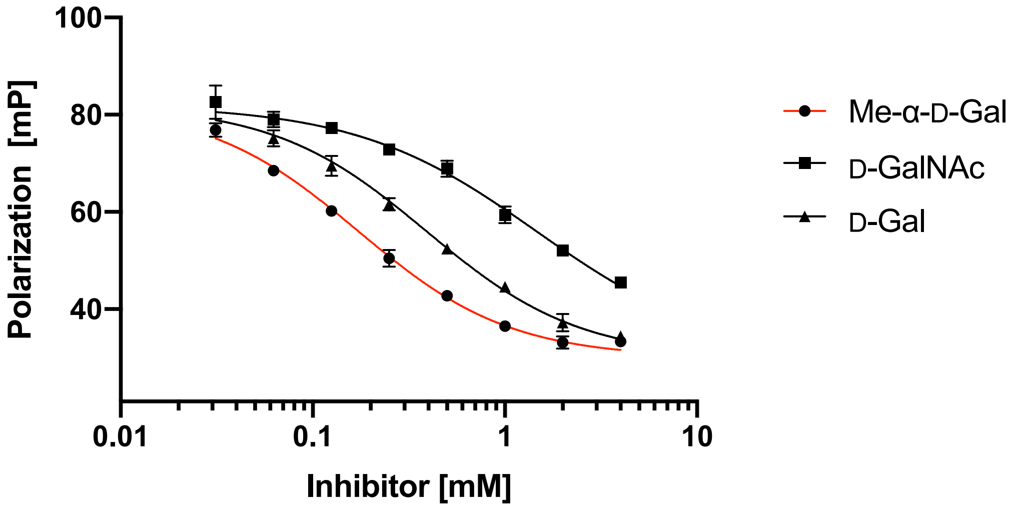

B

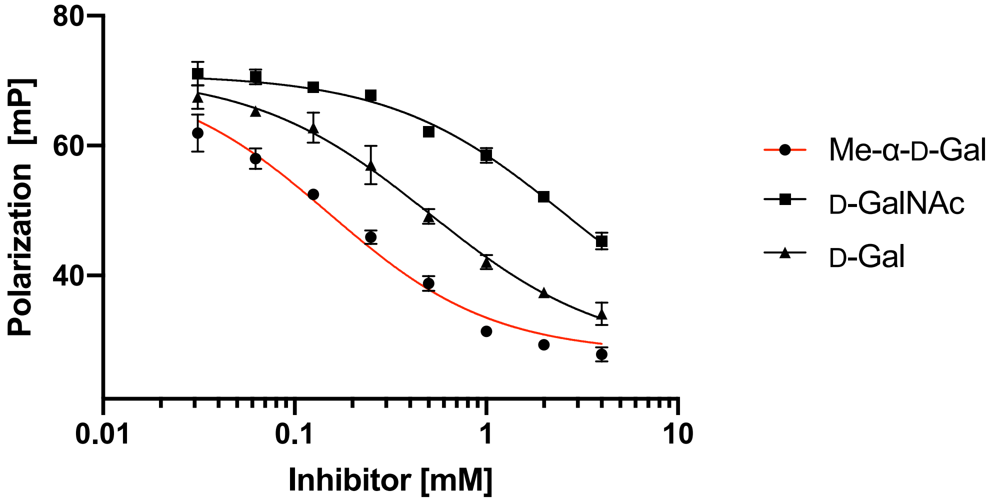

Fig. S7. Competitive binding assay based on fluorescence polarization with LecA and 5FW LecA. One representative titration of Me−α−D−Gal (positive control), D−GalNAc and D−Gal to **A.** LecA and **B.** 5FW LecA is depicted. IC_{50} values are given in **Table I** as average of three independent (LecA) or three technical replicates (5FW LecA).

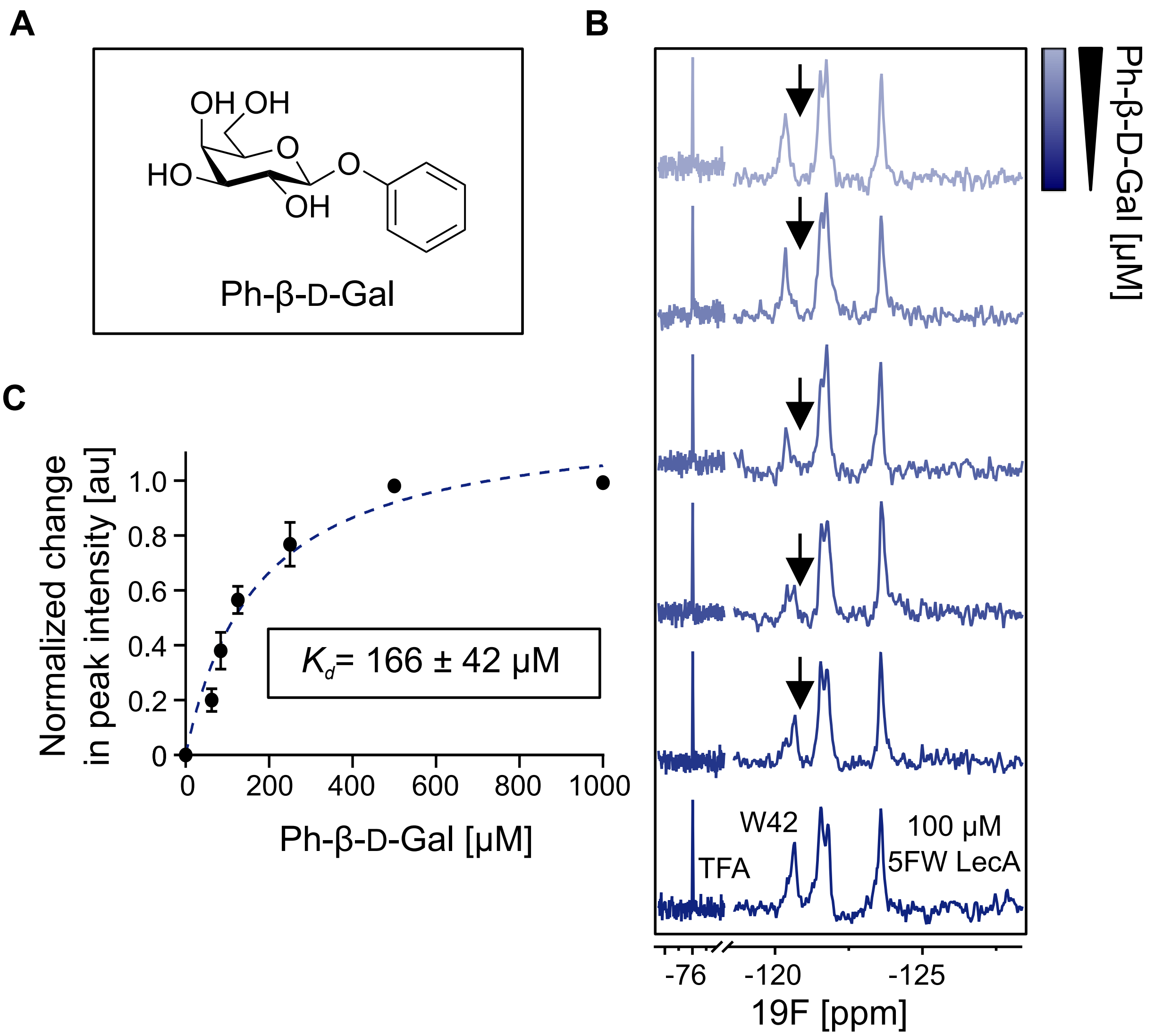

Fig. S8. PrOF NMR titration of Ph−β−D−Gal to holo 5FW LecA. **A.** The structure of Ph−β−D−Gal. **B.** The PrOF NMR titration of Ph−β−D−Gal to holo 5FW LecA in TBS pH 7.8 at 310 K. The W42 resonance from free protein appeared with decreasing intensity (*arrow*) upon Ph−β−D−Gal addition. The change in signal intensity of free W42 peak can be followed to determine K_d of 5FW LecA for Ph−β−D−Gal. **B.** Shown is normalized change in W42 peak intensity upon addition of Ph−β−D−Gal. For this, peak intensity in presence of Ph−β−D−Gal was dived by W42 peak intensity of the reference spectrum delivering the normalized change in peak intensity values plotted on Y-axis. Titration data was fitted to one−site−binding model to obtain K_d value of 166±42 μM. The error bars represent the standard deviation of three independent titrations.
