# Supplementary material for: Protein-observed 19F NMR of LecA from Pseudomonas aeruginosa
Source: Glycobiology. 2020 Jul 1;31(2):159–65. doi: 10.1093/glycob/cwaa057 (PMC7874386; doi:10.1093/glycob/cwaa057)
Supplement: GLYCO-2020-00049_Revision_cwaa057 [file glyco-2020-00049_revision_cwaa057.docx]

**Reviewer: 1**
*Comments to the Author*

*The manuscript of Shanina et al. entitle Protein-observed 19F NMR of LecA from ..." introduces an innovative application of protein-observed 19F (PrOF) NMR spectroscopy for monitoring and quantifying of lectin ligands. The advantages of the method are the simplicity of the measurement and that interactions with low affinity can be monitored.
However, there are some important details missing to fully judge the potential of PrOF NMR as a valuable tool for binding studies during the development of glycomimetics as drug-like inhibitors of lectins. The description of the approach could be improved.*

*Major points:*

*1. The idea behind the presented approach is brilliant and the presented data are very promising.
However, for establishing protein-observed 19F NMR as a reliable method to measure ligand affinities of lectins, every step of the procedure has to be transparent and convincing. 
Central to derive KD values are the plots that show a function of fraction bound versus the ligand concentration. The fraction of bound protein is in the manuscript somehow estimated by "% Δ (Intensity)" (in Figures 3, S2 and S3) and this part is not well described and thus not transparent. How is 100% defined?
Was the vanishing signal of the unbound protein used with 100% intensity at the beginning and 0% at the end? Or was just the signal of the bound form followed by starting with 0% and ending with 100% at a very large excess of the ligand?
Shown are only few data points and the last point is far from reaching saturation. I have the suspicion that 100% was defined as "total signal intensity" = "signal intensity of free protein" + "signal intensity of bound form". Such a definition would NOT be reliable and would introduce a systematic error of the KD values, because the line widths of both signals might be different and the signal intensity of the bound form might never reach the same intensity as the initial signal of the free form. The authors observed already that the line widths of the 19F signals depend on the protein concentration and on the temperature (Table S1). Therefore it is fair to assume that they also depend on the absence or presence of ligand. In addition, if the signal of the free form is partially overlapping with the signal of the bound form, it is very difficult to measure the exact intensities. For example at a concentration of 50% bound and 50% free two partially overlapped signals would show an intensity > 50% resulting in a steeper curve (intensity versus ligand concentration) suggesting a stronger binding.*

**Here, we followed changes in peak intensity of free protein W42 upon addition of the ligand. We apologize for not being clear and have now changed the label of Y-axis providing the normalized value 1.0 for W42 peak intensity in the reference spectrum (free protein without a ligand). We ensured that all spectra were processed and normalized in Mestrenova to the internal reference TFA at -75.6 ppm and 100 for the peak intensity.**

**Since unbound W42 peak intensity decreased upon addition of the ligand, we used these values to derive the normalize change in peak intensities following the equation (1) resulting in values plotted on Y-axis.**

$\boldsymbol{I}_{\boldsymbol{normilized}}\boldsymbol{=}\frac{\boldsymbol{I}_{\boldsymbol{0, reference}}\boldsymbol{-}\boldsymbol{I}_{\boldsymbol{measured}}}{\boldsymbol{I}_{\boldsymbol{0, reference}}}$ **(1),**

**where *I_0,reference_* is the W42 free peak in the reference spectrum (protein only), *I_measured_* is the W42 free peak in the spectrum protein with a ligand.**

**In addition, the integrals can be applied to derive Kd values, but it has limitations if free and bound peaks are partially overlapping. For that reason we used peak intensity to determine affinities.**

**We edited this information in the revised manuscript.**

- **Line 214**

*Can the errors of the data be estimated by error bars (in the y-axis of the plots)? The scattering seems to be quite large. In addition there might be a dilution effect due to the increase in volume.*

**We agree with the reviewer and introduced error bars for the titration replicates in all figures. The dilution effect was taken into account when we titrated the compound to it and it did not exceed 1% of volume having a minimal contribution to the dilution effect. The error bars are in range of maximum 10% which can depend on several factors: 1) differences in sample preparation, 2) pipetting error to the 3mm tube, 3) how well the compound was mixed with solution in a 3mm NMR tube, 2) phase change during recording NMR spectra, though we made sure that it is consistent during processing data in Mestrenova.**

*2. Comparing the given affinities of several ligands that are given in the Introduction with data available in publications is a bit confusing. This is mainly due to inconsistencies in the literature, but citing goes hand in hand with the responsibility to critically judge the data that is cited. 
There is a small discrepancy concerning the affinity between LecA and D-Gal: Kadam et al. 2011 reports a KD of 88 μM (cited) and Cioci et al. 2003 report 29 μM (Ka of 3.4x104 M-1). By looking into the original data of Kadam et al. 2011 (Figure S25) I noticed that the ITC data seem to be of poor quality. Whereas an ITC should to be planed such that the concentration of the protein and the amount of ligand is adequate to obtain a sigmoidal curve from which the stoichiometry can be determined, the ITC data of D-Gal in Kadam et al. 2011 starts with the first point already at approx. 1:1 stoichiometry. The protein concentration seems to be too low and during the titration too much ligand seems to be injected per point. Of course a KD can be calculated from such data using a fixed stoichiometry of 1:1, but the obtained values will not be very reliable.*

**We agree with the reviewer and indeed, there is a discrepancy in reported Kd values for galactose. We referred to Kadam, et al. 2011 because only they have reported the Kd using ITC. Turnbull et al., 2003 reported that the Kd value can be reliable even without a sigmoidal curve as long as the value of binding stoichiometry ‘*n*’ has been fixed. Moreover, the paper from Cioci et al. 2003 cited work from Garber et al. which used equilibrium dialysis instead of ITC making the comparison of affinities more difficult.**

**On this end, the Kd value for galactose from PrOF NMR deviated from previously reported Kd values. As we stated in the manuscript, the two− or three−fold deviations have been considered acceptable in PrOF NMR for affinity assessment (Gee, et al. 2016). Based on this publication, this range referred to weak (high µM – low mM range) ligands that rather promote fast exchange on the chemical shift timescale. Our ligands promote changes in slow exchange indicating much stronger binding. To best of our knowledge, application of PrOF NMR to derive affinities of strong binders has not been reported before and thus, we could not refer to a publication that defined what is an acceptable deviation range for strong binders. Based on performed titration experiments for this manuscript and reported Kd values for ligands used, we set a fourfold deviation from reported affinities to be acceptable for affinity assessment of strong binders in PrOF NMR.**

**Edits are in Line 123.**

*Please verify the Kd value for GalAG0 of 2.9 μM (line 50). Kadam (2011) reports 4.2 μM.*

**Line 45: We apologize and changed the value to 4.2 µM for *GalAG0.***

**Reference:**

**Kadam R U, Bergmann M, Hurley M, Garg D, Cacciarini M, Swiderska M A, Nativi C, Sattler M, Smyth A R, Williams P and others. A glycopeptide dendrimer inhibitor of the galactose-specific lectin LecA and of Pseudomonas aeruginosa biofilms. Angew Chem Int Ed Engl 2011;50(45):10631-5.**

**Turnbull WB, Daranas AH. On the Value of c:  Can Low Affinity Systems Be Studied by Isothermal Titration Calorimetry? Journal of the American Chemical Society 2003;125(48):14859-14866.**

**Garber N, Guempel U, Belz A, Gilboa-Garber N, Doyle R J. On the specificity of the D-galactose-binding lectin (PA-I) of Pseudomonas aeruginosa and its strong binding to hydrophobic derivatives of D-galactose and thiogalactose. Biochim Biophys Acta 1992;1116(3):331-3.**

**Gee C T, Arntson K E, Urick A K, Mishra N K, Hawk L M, Wisniewski A J, Pomerantz W C. Protein-observed (19)F-NMR for fragment screening, affinity quantification and druggability assessment. Nat Protoc 2016;11(8):1414-27.**

*3. In principle lectins can prefer one configuration of a monosaccharide, which is often the case. D-Gal adopts in aqueous solution ~31% the α-pyranose and to 62% the β-pyranose form and a much smaller amount of furanose forms (Zhu et al. 2001, J. Org. Chem. 66, 6244). The anomerization kinetics are likely slower than protein binding. For D-Gal the life times of the anomers seem not to be reported so far, but for D-Glc they were reported in the range of 10 min (Lewis et al. 2006, J. Am. Chem. Soc. 128, 5049-5058). There is a risk that affinities measured e.g. by ITC pick up only the binding of one conformer and calculating the KD using the total D-Gal concentration might distort the measured KD value. However, Rodrique et al. 2013 reported for LecA very similar KD values for α-D-Gal and β-D-Gal, namely 50.0 μM and 55.7 μM, respectively, which suggests that the anomeric configuration is not critical in this context (at least not for a small methyl group). However, if D-Gal is part of a larger glycan this might be different.*

**We agree with the reviewer and this should be clearly stated. In scope of this work we used D-Gal simply to check the activity of the protein and to ensure that it is not altered due to incorporation of 5FW. In general, we agree that using Me-α-D-Gal would have been a better choice. We included a sentence and the citation to highlight this fact in line 115.**

*4. There is another aspect of ligand binding that I missed: Blanchard et al. 2008 (J. Mol. Biol. 383, 837-852) reported glycan array data of LecA, which clearly showed that the glycans that bound best were oligosaccharides with a terminal D-Gal linked with an α-linkage to another D-Gal (mainly Galα1,3Galβ ... and  Galα1,4Galβ ...). Glycans with a terminal β-D-Gal are lacking among the top 20 hits (CFG primscreen_3381; PA-IL-0.1_12867_v4.1_DATA.xls), strongly suggesting that the preferred ligand contains a terminal α-D-Gal. This is further supported by the crystal structure of the PA-IL/Galα1,3Galβ1,4Glc complex (Blanchard et al. 2008) that shows also contacts of the second D-Gal to the protein. Imagining how a terminal β-D-Gal would be recognized: it cannot adopt the same orientation as in the crystal structure, since there is no space for the adjacent D-Gal. For some unknown reason all the cited references reporting improved/multivalent glycomimetics used terminal β-D-Gal moieties, which I would not consider ideal.*

**We thank the reviewer for bringing this to our attention. We introduced a clarification for β-linked D-Gal glycomimetics in the revised manuscript (Line 48).**

**Garber at al., 1992 has reported that α-Methyl-galactoside was a stronger inhibitor of LecA than the β-methyl derivative. However, the hydrophobic phenylated derivatives of the β-configuration of D-galactose were more potent inhibitors than the respective α-galactosides. For this reason, most of glycomimetics for LecA are designed in the β-configuration.**

**To visualize how LecA can adopt binding of β-linked D-galactopyranosides, (Kadam, et al. 2011) and (Rodrigue, et al. 2013) reported crystal structures of LecA with two low molecular weight glycomimetics, such as p-nitrophenyl β-D-galactoside (pNPGal, K_d_ = 14.1 µM, (Kadam, et al. 2011), pdb: 3ZYF) and naphthyl β-thio-D-galactoside (K_d_ = 6.3 µM (Rodrigue, et al. 2013) pdb: 4A6S). As result, the affinity of D-Gal has been improved around 6 to 7-fold upon addition of p-nitrophenyl or naphtyl rings in a β-linkage compared to galactose. Based on X-ray structures, this improvement is due to the additional CH−π interaction between the aromatic ring of the binding ligand and H50 at the binding pocket.**

**Additionally, we decided to confirm the observation for pNPGal and check if we can follow the gain in affinity compared to D-Gal using PrOF NMR. For this, we titrated pNPGal to 5FW LecA resulting in Kd of 54 µM p-nitrophenyl compared to D-Gal showing 360 µM affinity in PrOF NMR, which was a 6-fold improvement in binding affinity upon addition of p-nitrophenyl to D-Gal in the β-linkage.**

**This shows: 1) the gain in affinity for a pNPGal could be observed in PrOF NMR despite the deviation in reported affinities and 2) PrOF NMR can be used in design of glycomimetics.**

**Line 150: edits are in the revised version of the manuscript and supported with Figure 4.**

**References:**

**Kadam R U, Bergmann M, Hurley M, Garg D, Cacciarini M, Swiderska M A, Nativi C, Sattler M, Smyth A R, Williams P and others. A glycopeptide dendrimer inhibitor of the galactose-specific lectin LecA and of Pseudomonas aeruginosa biofilms. Angew Chem Int Ed Engl 2011;50(45):10631-5.**

**Rodrigue J, Ganne G, Blanchard B, Saucier C, Giguere D, Shiao T C, Varrot A, Imberty A, Roy R. Aromatic thioglycoside inhibitors against the virulence factor LecA from Pseudomonas aeruginosa. Org Biomol Chem 2013;11(40):6906-18.**

**Garber N, Guempel U, Belz A, Gilboa-Garber N, Doyle R J. On the specificity of the D-galactose-binding lectin (PA-I) of Pseudomonas aeruginosa and its strong binding to hydrophobic derivatives of D-galactose and thiogalactose. Biochim Biophys Acta 1992;1116(3):331-3.**

*4. For reproducibility reasons more experimental details concerning the NMR spectra are required, e.g. number of scans, recycle delay and measurement time.*

**We thank the reviewer for bringing this to our attention and included this information in the revised version of the manuscript.**

- **Line 199**

*5. line 100, "the line width of tryptophan resonances at 50 μM 5FW LecA concentration was too broad to result in a well resolved PrOF NMR spectrum (Table S1)". I cannot see a difference in line widths between 50 μM and 200 μM in Fig. S1. The signal-to-noise is just much smaller at 50 μM. From such a noisy spectrum (50 μM) I would not recommend to measure line widths.*

**We agree with the reviewer and did not measure line width as shown in Fig. S2 for 50uM because it of the low signal to noise. Only W84 as a single standing peak could be well resolved. Same applies to spectra at 285 K.**

*Table S1 is a bit puzzling. How reliable are the reported line widths? I am wondering which molecular mechanism could explain an increase in line width with increasing temperature? or first a decrease in line width followed by an increase (W33). Why would the line widths drop with increasing protein concentration (W2)? Unless the oligomerization state changes, which could influence the line widths, they should not depend on the protein concentration. However, increasing protein concentration would shift equilibrium towards higher oligomers and thus larger line widths. An increased viscosity would also lead to larger line widths with larger protein concentration, but not to sharper lines. 
Or are the differences in line widths just artifacts due to noisy spectra? Please re-evaluate this data and if they are shown In Table S1, I recommend adding all associated NMR spectra in the Supplementary Information as well.*

**We agree with the reviewer and have re-evaluated Table S1. Initially, peak line widths of tryptophan resonances were measured in MestReNova using Line Fitting function for automatic fitting of peaks (*red line*), which resulted in the deviation of line widths. As the reviewer pointed out, the spectra are noisy and thus, the automatic fitting of peaks does not work reliably. For this, we fitted peaks by adjusting the automatic fit manually (*red lines* *in* Fig. S1, S2, S3) reducing the deviation for W42 and W84 resonances. However, this could not be achieved as reliably for W2 and W33 due to a strong overlap of both resonances. To support this and help to understand the Table S1, we included corresponding spectra that have been used to determine line-widths of 5FW resonances in Mestrenova.**

*6. There is another advantage of using 5-fluorotryptophane that the authors did not mention so far:
tryptophan is by far the most frequently found amino acid in carbohydrate binding sites (see for example Taroni, Jones and Thornton 2000, Prot. Engin. 13, 89-98; or Hudson et al. 2015, J. Am. Chem. Soc. 137, 15152−15160). Although LecA does not directly involve Trp in the carbohydrate recognition, the fact that most lectins have a Trp in its binding site makes the method widely applicable.
However, in some cases the affinity might be changed due to the fluoro-substituent, in the examples of Hudson the interaction got stronger.*

**We thank the reviewer for bringing this information to our attention. We included both aspects and the references in the revised manuscript:**

- **Line 170**
- **Line 110**

*Minor points:

line 114, “W42 showed slow exchange on the NMR chemical shift time scale” is not really obvious from Figure 2E. In contrast, slow exchange is nicely visible during the titration with Ca2+ (Fig. S2).*

**Edits are in the revised version of the supplementary information in Fig. S4.**

*line 242, "Me− −D−Gal" there seems to be a Greek symbol missing*

**Edits are in the revised version of the manuscript.**

- **Line 245**

*Figure S3: the exact frequency (or frequencies), where the signal intensity was measured, should be indicated by a precise arrow, not a bold arrow*

**Edits are in the revised version of the supplementary information and this Figure has been renamed to Figure S5.**

**Reviewer: 2** *Comments to the Author*

*In this paper Authors carry on a proof of concept work to demonstrate that 19F NMR can be useful to probe the affinity and the epitope mapping of carbohydrate (derivatives) ligand to LecA, a pseudomonas protein which is crucial for its lethality. Authors incorporate the fluorine atom as 5-fluoro-tryptophan by adding to a recombinant E.coli strain the fluorinated indole precursor. They prove that this strategy is successful by mass spectrometry (However I trust figure 2 is useless, it is just an experimental protocol) and then produce the wt protein and its 4 triptophan mutants. Afterwards they assess the binding with calcium ion and gal and galNAc, this latter as weak ligand. All the binding is detected by 19F-NMR, the protein mutants help to assess which triptophan is closer and and this is also orthogonally counterproved by other biophysical approaches.
The work is rigorous and well conducted, a pity that authors didn’t try the binding of some real inhibitors but only two monosaccharide residues, reducing in this way the output of the work to a proof of concept with no other information; otherwise the paper is ok.*

**We thank the reviewer for a positive feedback and to support that our method is suitable for design of ligands for the carbohydrate-binding site of LecA, we performed two more PrOF NMR titrations using previously reported glycomimetics for LecA: pNPGal (Figure 4, Kadam et al., 2011) and this compound lacking a 4-nitro group on phenyl ring, phenyl-β-D-Gal (Figure S8, Garber et al., 1992). As result, p-nitrophenyl group in a β-linkage improved binding affinity of D-Gal 6-fold, which is in agreement with previous reports (Rodrigue et al., 2013). We believe that 5FW in LecA can serve as sensitive probes to design glycomimetics for the carbohydrate binding site of LecA. Finally, we are going to show the strength of this method to discover drug-like molecules in the future work.**

**Edits are in the revised version of the manuscript (line 150, Figure 4) and supplementary information (Figure S8).**

**References:**

**Kadam R U, Bergmann M, Hurley M, Garg D, Cacciarini M, Swiderska M A, Nativi C, Sattler M, Smyth A R, Williams P and others. A glycopeptide dendrimer inhibitor of the galactose-specific lectin LecA and of Pseudomonas aeruginosa biofilms. Angew Chem Int Ed Engl 2011;50(45):10631-5.**

**Garber N, Guempel U, Belz A, Gilboa-Garber N, Doyle R J. On the specificity of the D-galactose-binding lectin (PA-I) of Pseudomonas aeruginosa and its strong binding to hydrophobic derivatives of D-galactose and thiogalactose. Biochim Biophys Acta 1992;1116(3):331-3.**

**Rodrigue J, Ganne G, Blanchard B, Saucier C, Giguere D, Shiao T C, Varrot A, Imberty A, Roy R. Aromatic thioglycoside inhibitors against the virulence factor LecA from Pseudomonas aeruginosa. Org Biomol Chem 2013;11(40):6906-18.**

**Reviewer: 3** *Comments to the Author:*

*The authors present a smart NMR-based approach to monitor lectin interactions. The work essentially presents the methodology, which is focused on the use of F-Trp non natural amino acids to follow interactions by 19F-NMR. Conceptually, the approach has already been presented to the scientific community. However, to the best of my knowledge, it is the first time that its value is applied to the lectin field. The work is solid, well performed by highly competent scientists and it deserves publication. However, there are a few points that could be addressed to improve the final version:*

*The authors claim that this strategy can be useful to identify drug-like molecules with moderate affinity. However, they only show the interaction with GalNAc (not even the alpha and beta anomers, to assess specificity).*

**We thank the reviewer for a positive feedback and to show that our method can be used for design of ligands for the carbohydrate-binding site of LecA, we performed two additional PrOF NMR titrations with previously reported glycomimetics for LecA: pNPGal (Figure 4, Kadam et al., 2011) and this compound lacking a 4-nitro group on phenyl ring, Ph-β-D-Gal (Figure S8, Garber et al., 1992). Moreover, p-nitrophenyl group in a β-linkage improved binding affinity of D-Gal 6-fold, which is in agreement with the previous reports (Rodrigue et al., 2013). We believe that 5FW in LecA can serve as sensitive probes to design glycomimetics for the carbohydrate binding site of LecA. Finally, we are going to show the strength of this method to discover drug-like molecules in the future work.**

**Edits are in the revised version of the manuscript (line 150, Figure 4) and supplementary information (Figure S8).**

**References:**

**Kadam R U, Bergmann M, Hurley M, Garg D, Cacciarini M, Swiderska M A, Nativi C, Sattler M, Smyth A R, Williams P and others. A glycopeptide dendrimer inhibitor of the galactose-specific lectin LecA and of Pseudomonas aeruginosa biofilms. Angew Chem Int Ed Engl 2011;50(45):10631-5.**

**Garber N, Guempel U, Belz A, Gilboa-Garber N, Doyle R J. On the specificity of the D-galactose-binding lectin (PA-I) of Pseudomonas aeruginosa and its strong binding to hydrophobic derivatives of D-galactose and thiogalactose. Biochim Biophys Acta 1992;1116(3):331-3.**

**Rodrigue J, Ganne G, Blanchard B, Saucier C, Giguere D, Shiao T C, Varrot A, Imberty A, Roy R. Aromatic thioglycoside inhibitors against the virulence factor LecA from Pseudomonas aeruginosa. Org Biomol Chem 2013;11(40):6906-18.**

*No comparison with ligand-based NMR techniques is performed. A sentence or paragraph would be acknowledged. For instance, competition experiments are more difficult to design with the presented methodology (protein observed method) than with STD-NMR.*

**We agree with the reviewer and added a sentence to acknowledge STD NMR the revised manuscript (Line 162).**

*The section devoted to the study and affinity estimation could be presented with a higher level of detail, given that it is the key asset of the work.*

*Under the titration conditions, the system enters into the slow exchange regime in the chemical shift time scale. Comments on this point would be highly appreciated and the exact description on how affinities were estimated. NMR signals for free and bound 19F-NMR signals show up in the spectrum. Therefore, a correlation between both peaks should exist with a constant sum value. Only four points are employed for the titrations. A comment on this small number would also be given.*

**We agree with the reviewer and introduced changes in the manuscript:**

1. **We added more data points to all our PrOF NMR titration data (Fig 3, S4, S5) in the revised manuscript and supplementary information. Notably, we did not observe a discrepancy compared to the Kd values we reported previously.**
2. **We introduced a description of how the affinities were estimated (Line: 216).**

**Briefly, we followed changes in peak intensity of W42 free form. To derive the *K_d_* value, we plotted a normalized change in peak intensity against the ligand concentration. The normalized change in peak intensity value was derived based on eq. (1):**

$\boldsymbol{I}_{\boldsymbol{normalized}}\boldsymbol{=}\frac{\boldsymbol{I}_{\boldsymbol{0}}\boldsymbol{-}\boldsymbol{I}_{\boldsymbol{measured}}}{\boldsymbol{I}_{\boldsymbol{0}}}$ **(1),**

**where *I_0_* is defined as W42 free form resonance in the reference spectrum of protein only, *I_measured_* is W42 free form resonance in the presence of protein with a ligand. The *K_d_* values were calculated according to the one−site−binding model in Graphpad PRISM 8.0.**

*Table 1 compares IC50 with Kd values. This should be explicitly stated, also describing that both numbers do not mean the same.*

**We thank the reviewer for this suggestion and clarified this in revised version. Table 1 has been moved to the supplementary information (Table SIV).**

*The figures should present the exact concentration of lectin employed. Overall, this is an excellent example of the power of NMR techniques to monitor glycan interactions.*

**We thank the reviewer for this suggestion. We added the information on concentration of protein (100 µM 5FW LecA) being used for PrOF NMR titrations for all figures in the revised manuscript and supplementary information.**

*Since ITC measurements are given, a comment on the obtained thermodynamic parameters would also very highly valuable. How is the enthalpy/entropy balance modified? Aromatic-pyranose stacking is probably not essential in this example, but this could be the case in other examples.*

**We cannot elaborate on this, because we do not compare two systems directly here and nothing has been reported on metal titration with LecA.**
